# Supplementary material for: Computational screening of high-performance optoelectronic materials using OptB88vdW and TB-mBJ formalisms
Source: Sci Data. 2018 May 8;5:180082. doi: 10.1038/sdata.2018.82 (PMC5944908; doi:10.1038/sdata.2018.82)
Supplement: Supplementary Information [file sdata201882-s2.docx]

**Supplementary** **Material: Computational screening of high-performance optoelectronic materials using OptB88vdW and TB-mBJ formalisms**

Kamal Choudhary^1^, Qin Zhang^2^, Andrew C. E. Reid^1^, Sugata Chowdhury^2^, Nhan Van Nguyen^2^, Zachary Trautt^1,3^, Marcus W. Newrock^3^, Faical Yannick Congo^1^, Francesca Tavazza^1^

1 Materials Science and Engineering Division, National Institute of Standards and Technology, Gaithersburg, Maryland 20899, USA

2 Physical Measurement Laboratory, National Institute of Standards and Technology, Gaithersburg, Maryland 20899, USA

3 Office of Data and Informatics, National Institute of Standards and Technology, Gaithersburg, Maryland 20899, USA

Table S1**:** Bandgap results and total computational time normalized by number of computer cores for calculations and total number of k-points using OPT, MBJ and HSE06 methods.

| Material | ID | OPT | | MBJ | | HSE06 | |
| --- | --- | --- | --- | --- | --- | --- | --- |
|  |  | Eg (eV) | Time (sec) | Eg (eV) | Time (sec) | Eg (eV) | Time (sec) |
| Si | JVASP-1002 | 0.73 | 0.12 | 1.28 | 0.43 | 1.22 | 20.03 |
| MoS_2_ | JVASP-54 | 0.92 | 0.73 | 1.34 | 26.6 | 1.49 | 112.4 |
| WS_2_ | JVASP-72 | 0.72 | 1.25 | 1.51 | 16 | 1.60 | 111 |
| GaS | JVASP-143 | 1.5 | 2.5 | 2.45 | 40.05 | 2.32 | 360 |
| BN | JVASP-17 | 4.46 | 0.22 | 6.1 | 1.6 | 5.5 | 16.03 |
| MoTe_2_ | JVASP-60 | 0.76 | 1.17 | 1.0 | 31.0 | 1.19 | 218.05 |
| MoSe_2_ | JVASP-57 | 0.91 | 0.56 | 1.32 | 18.8 | 1.40 | 138.8 |
| WSe_2_ | JVASP-75 | 1.05 | 0.8 | 1.44 | 17 | 1.52 | 98 |

We observe that computational cost for MBJ is an order of magnitude higher than OPT, while HSE06 computational cost is an order of magnitude higher than MBJ.


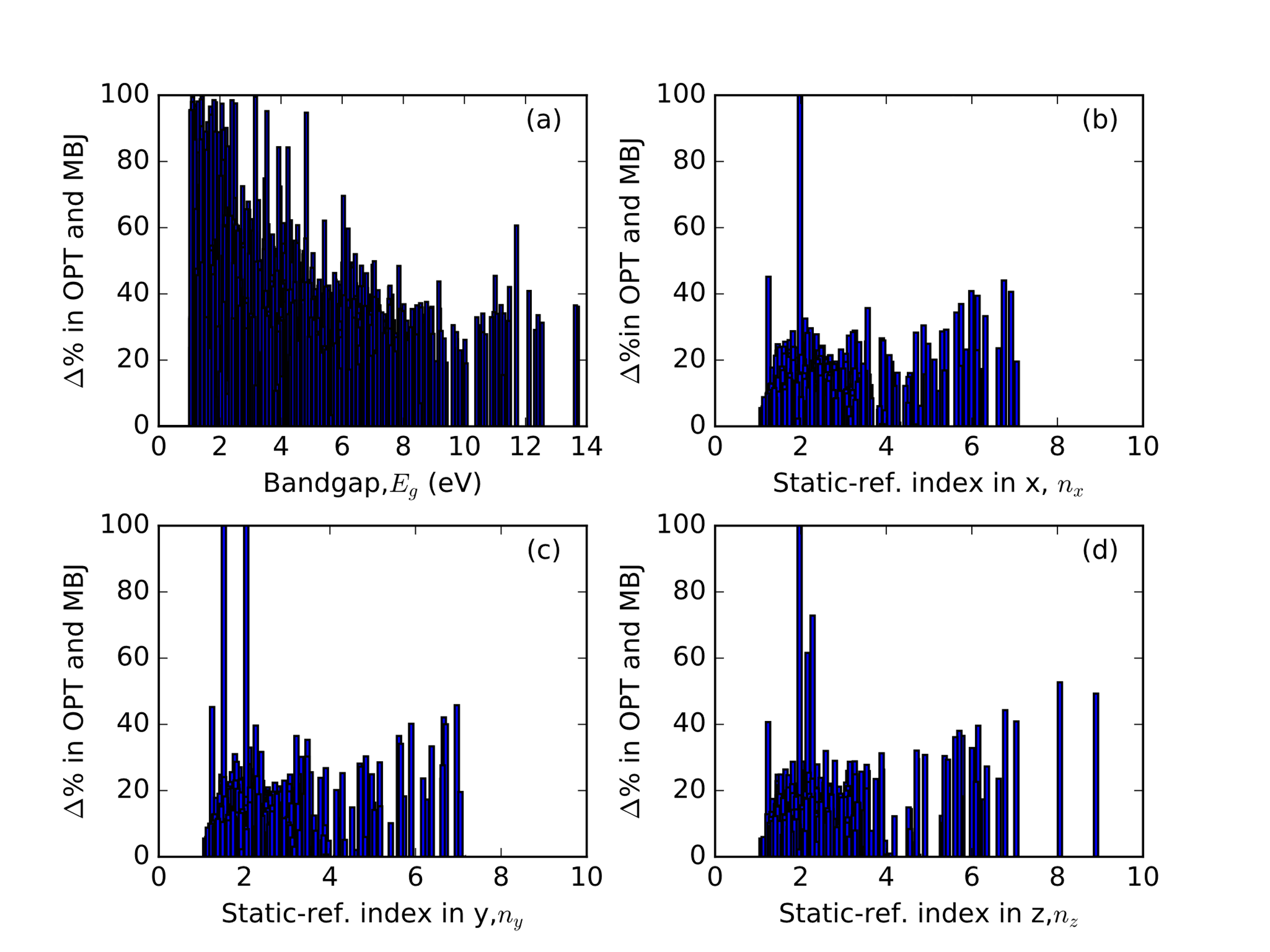


Fig. S1 Relative percentage difference in bandgap and static refractive indices between OPT and MBJ formalisms. a) bandgaps, b) static refractive index in c) static refractive index in x-direction, d) static refractive index in y-direction and d) static refractive index in z-direction.
